# Large-scale, prospective observational study of regorafenib in Japanese patients with advanced gastrointestinal stromal tumors in a real-world clinical setting

**DOI:** 10.3389/fonc.2024.1412144

**Published:** 2024-06-17

**Authors:** Yoshito Komatsu, Kei Muro, Masayuki Chosa, Kazufumi Hirano, Toshiyuki Sunaya, Koichi Ayukawa, Kana Hattori, Toshirou Nishida

**Affiliations:** ^1^ Department of Cancer Chemotherapy, Hokkaido University Hospital Cancer Center, Sapporo, Japan; ^2^ Department of Clinical Oncology, Aichi Cancer Center Hospital, Nagoya, Japan; ^3^ PMS, Bayer Yakuhin Ltd., Osaka, Japan; ^4^ Clinical Statistics, Bayer Yakuhin Ltd., Osaka, Japan; ^5^ Medical Affairs Oncology, Bayer Yakuhin Ltd., Osaka, Japan; ^6^ Japan Community Heathcare Organization, Osaka Hospital, Osaka, Japan

**Keywords:** regorafenib, gastrointestinal stromal tumor, observational study, post-marketing study, safety, effectiveness, Japanese patients

## Abstract

**Background:**

Regorafenib improves overall survival (OS) of patients with advanced progressive gastrointestinal stromal tumors (GISTs) after standard chemotherapy in phase III trials in the 3rd-line setting. This large-scale, prospective observational study evaluated the safety and effectiveness of regorafenib in Japanese patients with GIST in a real-world clinical setting.

**Methods:**

Patients with GIST received oral regorafenib at a maximum daily dose of 160 mg for weeks 1–3 of each 4-week cycle (dose could be modified at investigator’s discretion). The primary objective was to assess safety, particularly significant adverse drug reactions (ADRs), as well as the frequency of occurrence of ADRs, hand and foot syndrome (HFS), discontinuation of treatment due to disease progression and adverse events. A Cox proportional hazards model was used to evaluate associations between OS or time to treatment failure (TTF) and baseline characteristics or HFS.

**Results:**

Between August 2013 and March 2021, 143 evaluable patients were enrolled. ADRs occurred in 90.2% of patients and led to treatment discontinuation in 28.3%. The most frequent ADRs were HFS, hypertension, and liver injury. The overall response rate was 11.3% and disease control rate 56.5% (RECIST) based on investigators’ assessments. Median OS was 17.4 months (95% CI 14.24–23.68). Median TTF was 5.3 (95% CI 4.0–6.5) months. Improved OS and TTF responses occurred in patients with an Eastern Cooperative Oncology Group performance status (ECOG-PS) of 0 or 1.

**Conclusion:**

The outcomes in this real-world study were consistent with those seen in clinical trials. No new safety concerns were identified.

**Clinical trial registration:**

https://clinicaltrials.gov, identifier NCT01933958.

## Introduction

Gastrointestinal stromal tumors (GIST) are the most common soft tissue sarcomas of the gastrointestinal tract (1). The incidence of GIST in Japan has been estimated at 1500–2500 cases per year (2).

Oncogenic gain-of-function mutations of the genes encoding KIT and platelet-derived growth factor receptor A (PDGFRA) are the driver events for most GISTs, with activating *KIT* mutations present in approximately 80% of GISTs and *PDGFRA* mutations in 5–10% (3, 4). Tyrosine kinase inhibitors (TKIs) are the mainstay of the treatment of advanced GISTs. Imatinib, which inhibits signaling by KIT and PDGFRA, is generally used as first-line therapy, while sunitinib, which inhibits vascular endothelial growth factor receptors (VEGFRs) as well as KIT and PDGFRA, is used as second-line therapy (1, 3).

In the event of disease progression due to the development of resistance to both these drugs, the multikinase inhibitor regorafenib is commonly used as third-line therapy (1, 3). Regorafenib has activity against tyrosine kinases involved in oncogenesis (e.g., KIT, RET, and V600E-mutated BRAF), stromal maintenance (e.g. PDGFR and fibroblast growth factor receptor), angiogenesis (e.g. VEGFR 1–3 and TIE2) (3, 5), and tumor immunity (6). Its efficacy was demonstrated in the phase 3 GRID trial, in which it significantly improved progression-free survival (primary endpoint) and the disease control rate (DCR) compared with placebo in patients with metastatic and/or unresectable GIST that had progressed after treatment with imatinib and sunitinib (7).

The efficacy and safety profile in the small subgroup of Japanese patients who received regorafenib in GRID (n=12) was consistent with the overall study population of global patients (2). However, clinical trials involve carefully selected patients, which may not reflect the more heterogeneous population seen in routine clinical practice. Large-scale studies in a real-world setting are usually used importantly to confirm the effectiveness of a drug and to detect uncommon but clinically significant adverse drugs reactions (ADRs). Consequently, a large-scale multicenter observational post-marketing surveillance study was undertaken to investigate the safety and effectiveness of regorafenib under real-world conditions in Japan.

## Materials and methods

This prospective observational study involved 101 centers in Japan. The study (ClinicalTrials.gov, NCT01933958) was conducted in compliance with the Good Post-Marketing Study Practice (GPSP) and Good Vigilance Practice (GVP) of the Ministry of Health, Labor, and Welfare in Japan. Approval by the ethics committee of each institution was not required because GPSP and GVP do not require such approval for Post Marketing Surveillance (PMS) studies.

Patients with GIST in whom progression had occurred after receiving standard chemotherapy of imatinib and sunitinib, and for whom regorafenib treatment was planned, were registered centrally. Patients were excluded if they had severe liver injury [aspartate aminotransferase (AST) or alanine aminotransferase (ALT) >5 times the upper limit of normal (ULN), bilirubin >2.0 times the ULN], uncontrolled hypertension, or Eastern Cooperative Oncology Group (ECOG) performance status (PS) ≥2.

The recommended dose of regorafenib was 160 mg per day for 3 weeks, with 1 week off, as described in the GRID trial (7). The dose could be modified in accordance with the product label (8) and at the investigators’ discretion.

The main objectives of the study were to assess the safety (including the occurrence of unknown and clinically significant adverse drug reactions, ADRs) and effectiveness of regorafenib in real-world clinical practice. An ADR was defined as an adverse event for which a causal relationship with regorafenib could be likely.

Data were collected on demographic and disease characteristics, concomitant medications, regorafenib dose, adverse events, and laboratory values. Adverse events were summarized based on the medical dictionary for regulatory activities (MedDRA)/J, version 24.0 terminology. The severity of adverse events was graded using Common Terminology Criteria for Adverse Events version 4.0. The documentation of any AE/SAE ended with completion of the observational period of the patient. However, any AE/SAE – regardless of relationship and seriousness – occurring up to 30 days after the last dose of regorafenib within the study period had to be documented. Patients were followed up for 6 months for ADRs, serious adverse events, and serious ADRs, and for 3 years for overall survival (OS), time to treatment failure (TTF), and tumor response. Only deaths were reported in safety-related events after 6 months post-dose and later. The best overall response was classified as complete response (CR), partial response (PR), stable disease (SD), progressive disease (PD), or undeterminable, according to RECIST guideline version 1.1 or by clinical evaluation to assess tumor progression.

Statistical analyses were performed using SAS version 9.2 (SAS Institute Inc., Cary, NC). Given the exploratory nature of the study, all p values were nominal and were considered descriptive. Demographic characteristics were summarized descriptively. OS and TTF were estimated using the Kaplan-Meier method. OS was defined as the time from the date of first administration of regorafenib to the date of death (regardless of the cause) or censored on the last confirmed date of survival. TTF was defined as the time from the date of first administration of regorafenib to the date of treatment discontinuation for any reason including disease progression, adverse event, patient preference or death. Associations between OS or TTF and baseline characteristics or HFS were evaluated using the Cox proportional hazards model.

The incidence proportion of an abnormal liver function test (AST or ALT) over 3 times the upper limit of normal in the GRID trial was more than 5% (7). To detect at least 3 events at a 5% incidence proportion with 95% probability required a total of 125 patients for analyses. The target number of patients was set at 135 in our study, to account for a dropout proportion similar to those observed in other PMS studies.

## Results

**Figure 1:**
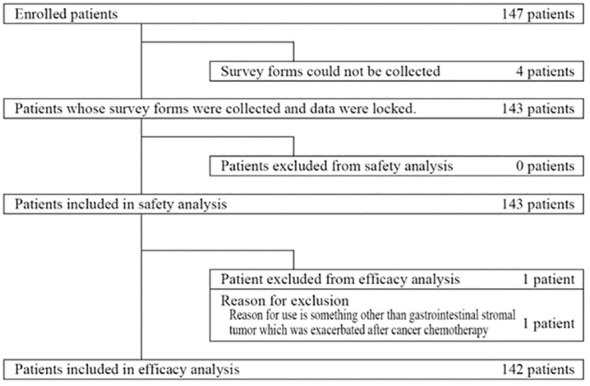
Disposition of patients.

Between August 2013 and March 2021, 147 patients were enrolled (**Figure 1**). Four patients were excluded because survey forms could not be collected. All remaining 143 patients were included in the safety analysis. The effectiveness analysis included 142 of these patients, after one patient was excluded because he received regorafenib for a cancer other than GIST.

### Patient characteristics

Baseline characteristics are summarized in **Table 1**. Among the 143 patients, 65.7% were men, the median age was 68 (range 20–86) years, and the median body mass index was 21.1 (range 14.9–29.1) kg/m^2^. One hundred and forty-two patients (99.3%) had GIST as the primary disease, while one patient had a malignant neoplasm with an unknown primary tumor. The most common sites for the primary lesion were the stomach (41.3%) and the small intestine (36.4%). The primary lesions had been resected in 81.8% of patients. Most patients (97.9%) had developed metastatic/recurrent lesions, which mainly occurred in the liver (66.4%) and peritoneum (57.3%). The median time from the date of disease progression to the date of first regorafenib administration was 22 (range, 1–377) days. Regorafenib was most commonly administered as third-line therapy (84.6%). All patients received chemotherapy as previous treatment, and the main drug used was imatinib in 98.60% (141/143) and sunitinib in 92.31% (132/143). Most patients had an ECOG performance status (ECOG-PS) of 0 (49.0%) or 1 (46.2%).

**Table 1:** Patient characteristics (n=143).

Characteristic	
Age (years), median (range)	68 (20–86)
Sex (male/female), n (%)	94 (65.7)/49 (34.3)
Body weight (kg), median (range)	54.4 (36.9–89.0)
Body mass index (kg/m^2^), median (range)	21.1 (14.9–29.1)
Primary disease, n (%)	
GIST progression after chemotherapy	142 (99.3)
Cancer of unknown primary	1 (0.7)
Primary lesion, n (%)	
Esophagus	2 (1.40)
Stomach	59 (41.3)
Duodenum	16 (11.2)
Small intestine	52 (36.4)
Large intestine	6 (4.20)
Outside of digestive tract	7 (4.90)
Stomach and outside of digestive tract	1 (0.70)
Tumor resection, n (%)	
Resected	117 (81.8)
Not resected	23 (16.1)
Unknown	3 (2.1)
Metastatic/recurrent lesions present, (%)	140 (97.9)
Local	16 (11.2)
Liver	95 (66.4)
Peritoneum	82 (57.3)
Lung	12 (8.4)
Bone	7 (4.9)
Other	15 (10.5)
ECOG performance status, n (%)	
0	70 (49.0)
1	66 (46.2)
2	5 (3.5)
3	2 (1.4)
4	0
Time from disease progression to start of regorafenib (days), median (range)	22 (1–377)
Line of therapy (regorafenib), n (%)	
First	0
Second	8 (5.6)
Third	121 (84.6)
Fourth	11 (7.7)
Fifth	0
Other	3 (2.1)

### Regorafenib treatment

The initial daily dose of regorafenib was 160 mg (standard dose) in 42.0% of patients, 120 mg in 34.3% of patients and 80 mg in 23.8% of patients.

**Figure 2:**
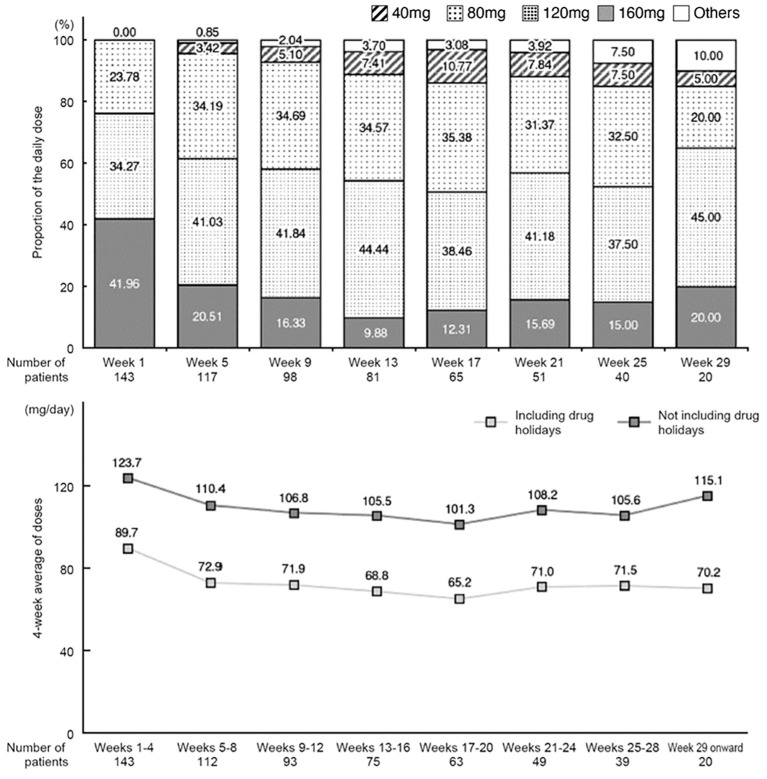
Change in the mean of daily dose (proportion relative to the number of patients on the first day of each period) and change in daily dose averaged over 4-week periods (including or excluding drug holidays).

Changes in the mean daily dose and changes in dose averaged over 4-week periods are shown in **Figure 2**. In week 1, a high proportion of patients received a mean daily dose of 120 mg or 160 mg, whereas in weeks 5–25, a high proportion received 80 mg or 120 mg (**Figure 2**). The mean daily dose tended to decrease over time up to week 17. After week 29, the number of patients was small (n=20); the daily dose was 120 mg in 9 patients, 160 mg in 4 patients, and 80 mg in 4 patients.

The 4-week average daily dose (excluding drug holidays) was 123.7 mg/day in the first 4 weeks and then decreased to 110.4 mg/day in weeks 5–8. It tended to decrease further in subsequent periods (average of 101.3–108.2 mg/day) through week 28, and then increased again from week 29 onwards (115.1 mg/day).

### Treatment discontinuation with regorafenib

Regorafenib therapy was discontinued in 81 (56.6%) patients as a result of cancer progression (52/81, 64.2%), adverse events (31/81, 38.3%) and ‘other’ (7/81, 8.64%) [some patients had more than one reason] (**Supplementary Figure 9**). Among the reasons for treatment discontinuation, adverse events were more common in the first 4 weeks, and discontinuation due to disease progression of cancer was more common after Week 5.

The percentage of patients who discontinued treatment due to adverse events was 63.2% (12/19) in weeks 1 to 4, 16.7% (2/12) in weeks 5 to 8, 7.7% (1/13) in weeks 9–12, 27.3% (3/11) in the weeks 13–16, 12.5% (1/8) in the weeks 17 to 20, 16.7% (1/6) in the weeks 21 to 24, and 16.7% (1/6) in the weeks 25 to 28.

### Safety

ADRs occurred in 90.2% of 143 patients included in the safety analysis (**Table 2**). The most common ADRs were HFS (63.6%), hypertension (32.2%), abnormal hepatic function (15.4%), and malaise (14.0%).

**Table 2:** Adverse drug reactions (ADRs) reported for ≥5% of patients treated with regorafenib (n=143).

	Any grade, n (%)
Any ADR	129 (90.2)
Hand and foot syndrome	91 (63.6%)
Hypertension	46 (32.2%)
Hepatic function abnormal	22 (15.4%)
Malaise	20 (14.0%)
Decreased appetite	14 (9.8%)
Dysphonia	13 (9.1%)
Platelet count decreased	13 (9.1%)
Hypothyroidism	10 (7.0%)
Diarrhea	9 (6.3%)
Neutrophil count decreased	9 (6.3%)
Stomatitis	8 (5.6%)
Pyrexia	8 (5.6%)

There appeared to be a tendency for the incidence of ADRs overall, and the incidence of hepatic function disorders and HFS (priority ADRs in this survey), and fatigue/malaise (ADRs of special interest) to increase with increasing initial daily dose of regorafenib; however, 95% confidence intervals for the proportions at each dose level overlapped (**Supplementary Table 1**; **Supplementary Figures 3–6**).

Overall ADRs, and ADRs graded ≥3, were observed more frequently during the early period of regorafenib administration (up to 60 days), and the incidence did not increase beyond 182 days after the start of treatment (**Supplementary Figure 1**). Similar trends were seen when ADRs of special interest (hepatic function disorders, hypertension/hypertensive crisis, HFS, and fatigue/malaise) were analyzed separately (**Supplementary Figures 2–6**).

### Effectiveness

Among patients in whom the best overall tumor response was evaluated according to RECIST (n=115), the response rate (PR or better) was 11.3% (13/115 patients), the disease control rate (DCR; SD or better) was 56.5% (65/115), and 40.9% (47/115) had PD. Regarding the 10 patients in whom the best overall tumor response was evaluated by investigators clinically with other methods instead of RECIST, the response rate (PR or better) was 70.0% (7/10 patients), and the DCR (SD or better) was 100.0%. Seventeen patients for whom the evaluation method or result was not recorded were excluded from the analysis of best overall response (**Table 3**).

**Table 3:** Best overall response according to RECIST.

Number of patients assessed	CR	PR	SD	PD	Could be not assessed	Response rate [95% confidence interval]	Disease control rate [95% confidence interval]
115	0	13	52	47	3	11.30% [6.16–18.55]	56.52% [46.96–65.74]

**Figure 3:**
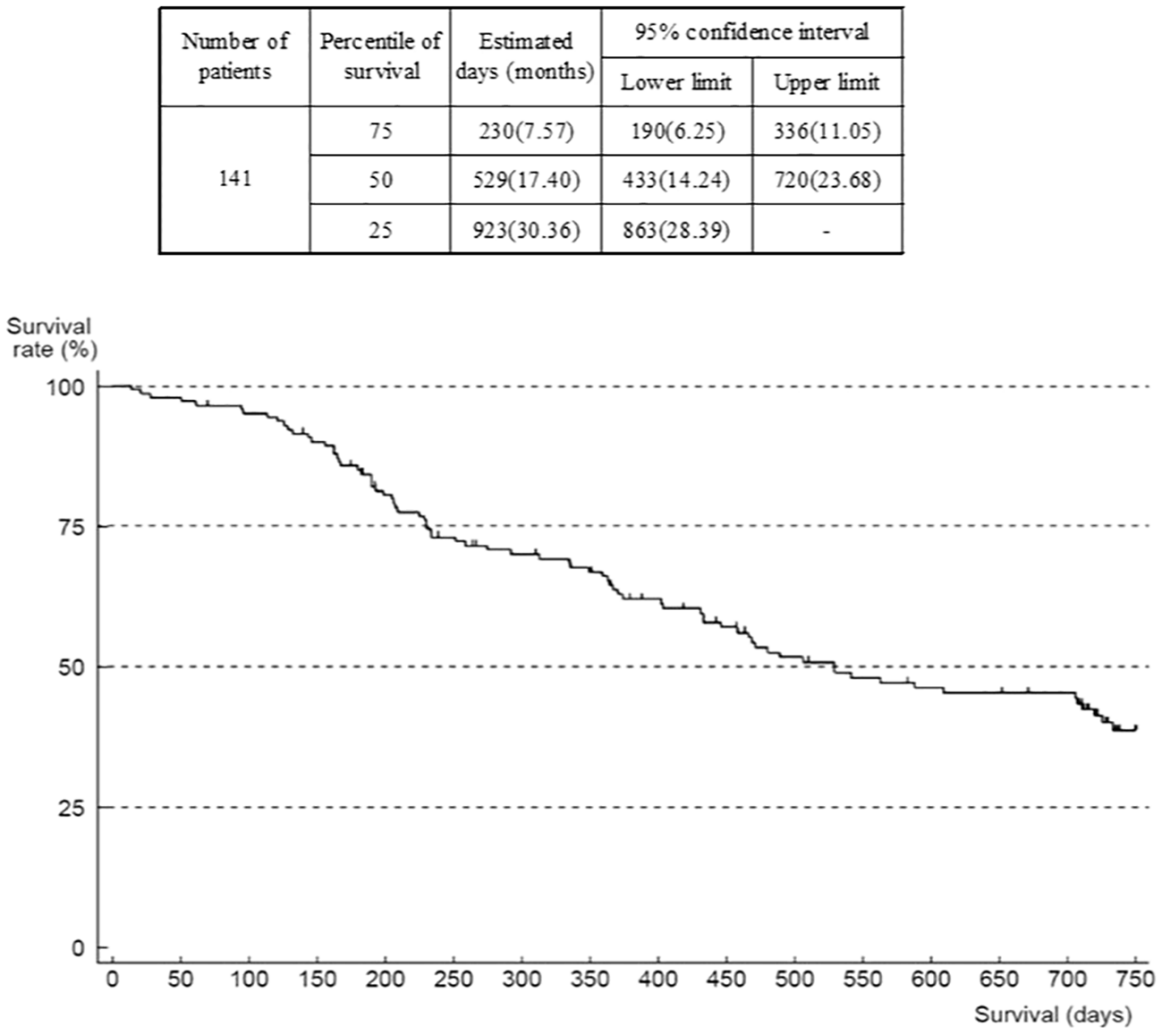
Overall survival (all patients).

The outcome was death in 80 patients, mostly due to progression of the primary disease (GIST). Median OS was 17.4 months (95% confidence interval (CI) 14.24–23.68) (**Figure 3**). The number of patients in OS and TTF analyses was 141 (of 142) because there was one patient with missing data on the date of last survival confirmation or death that was censored. When analyzed according to ECOG-PS at the start of regorafenib administration, median OS was 30.4 (95% CI 23.85–not reached) months in the group of patients with an ECOG-PS of 0, compared with 12.2 (95% CI 7.70–15.33) months in the group with ECOG-PS of 1, and 5.4 months (95% CI 0.69–not reported) in those with an ECOG-PS of ≥2 (**Supplementary Figure 7**). The Cox univariate model indicated that the hazard ratio of the ECOG-PS 0 group versus the ECOG-PS 1 group was 3.68 (95% CI 2.24–6.04), and versus the ECOG-PS ≥2 group was 3.77 (95% CI 1.30–10.96). However, this comparison is not deemed reliable because of the low number of patients with ECOG-PS ≥2. There is a trend that patients with worse ECOG-PS at the start of administration of study treatment were more likely to have shorter survival.

When analyzed according to the worst grade of HFS experienced, median OS was 15.4 (n=51, 95% CI 7.53–23.85) months in the group who had no HFS, 23.3 (n=19, 95% CI 11.9–not reported) months in the HFS grade 1 group, 17.8 (n=48, 95% CI 13.2–not reported) months in the HFS grade 2 group, and 16.6 (n=22, 95% CI 11.8–not reported) months in the HFS grade 3 group. Hazard ratios of the no-HFS group versus groups with HFS were 0.64 (95% CI 0.32–1.32) versus the HFS grade 1 group, 0.72 (95% CI 0.42–1.21) versus the HFS grade 2 group, and 0.63 (95% CI 0.32–1.25) versus the HFS grade 3 group, indicating that there was no significant difference in survival between patients with no HFS and those with HFS of any grade.

**Figure 4:**
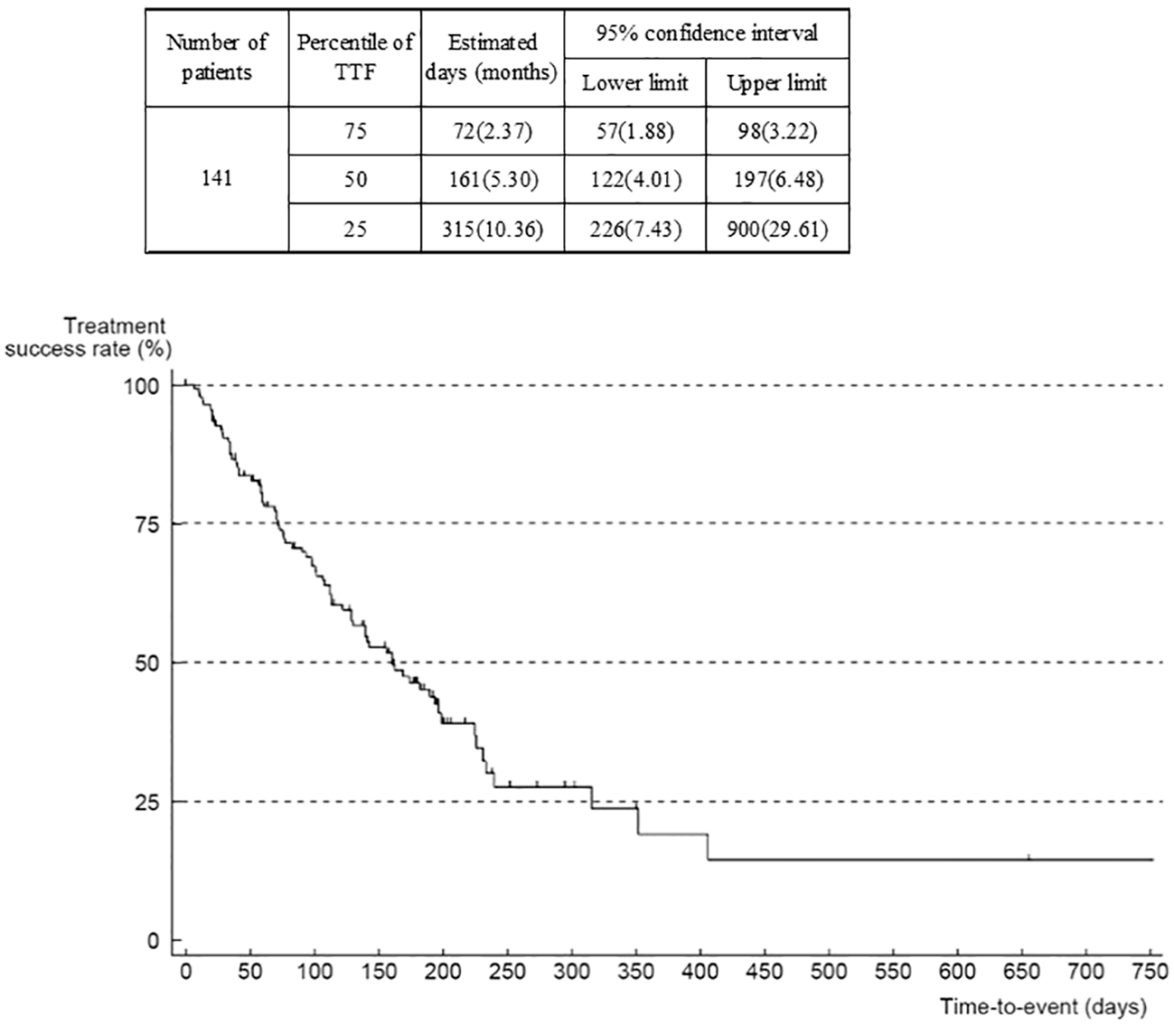
Time to treatment failure (all patients).

Median TTF was 5.3 (95% CI 4.0–6.5) months (**Figure 4**). When analyzed according to ECOG-PS at the start of regorafenib administration, median TTF was 6.6 (95% CI 5.3–11.6) months in the group whose ECOG-PS was 0, compared with 4.2 (3.2–6.0) months in the group whose ECOG-PS was 1, and 3.7 months in the group whose ECOG-PS was ≥2 (**Supplementary Figure 8**). The hazard ratio of the ECOG-PS 0 group versus the ECOG-PS 1 group was 1.60 (95% CI 1.00–2.56), and versus the ECOG-PS ≥2 group was 2.02 (0.70–5.83), suggesting that the worse the ECOG-PS was at the start of regorafenib administration, the shorter the TTF. The median TTF by metastatic or recurrent site was 5.13 (95% CI 3.22–7.66) months for liver only, 5.36 (95% CI 3.22–7.40) months for peritoneum only, and 5.30 months for peritoneum plus 1 organ indicating that there were no substantial differences between metastatic or recurrent sites.

## Discussion

This paper reports the results of an observational post-marketing surveillance study investigating the safety and effectiveness of regorafenib in Japanese patients with GIST treated in routine clinical practice. There were no deaths due to severe liver injury identified as being related to regorafenib. In the previously published GRID study, severe liver disfunction occurred in a 49-year-old Japanese man with metastatic GIST who discontinued regorafenib treatment as a result of acute hepatic failure that was considered to be drug related. The patient was hospitalized 9 days after beginning his second cycle of treatment with symptoms of hepatic failure, and regorafenib was discontinued; the patient died 2 weeks later (2). Consequently, further enrollments were suspended and only 17 Japanese patients had. As a result of this death, and a requirement for the regulatory approval of regorafenib for GIST in Japan, weekly liver function testing is recommended during the first 2 months following administration of regorafenib. This allows physicians to confirm liver function abnormalities early and manage them appropriately by, for example, discontinuing the drug. No further deaths attributable to regorafenib have been reported. Additionally, there were fewer liver/hepatic function abnormalities with appropriate handling including dose reductions and drug holidays. This is a good example how experience in clinical trials is utilized to improve clinical practice.

In the current study, dose reductions were allowed to manage ADRs, which tended to occur more frequently at higher initial dose levels. The incidence of ADRs increased primarily in the first 60 days (8.6 weeks) after the start of observation and the number of patients treated with regorafenib gradually decreased. Overall, treatment was discontinued due to ADRs in 31 patients (21.7%). Appropriate management of ADRs with regorafenib is now well established, and physicians are more familiar with its benefit: risk ratio, resulting in continuation of treatment in many cases. Subsequently, at 182 days (26 weeks) after the start of treatment, the number of patients who continued regorafenib administration decreased because of disease progression and adverse events. These results highlight the importance of monitoring adverse events in the early period of treatment with regorafenib and managing them with appropriate dose modification or drug holidays, in order to minimize the number of patients who need to permanently discontinue treatment. In most medical institutions, liver function is monitored regularly. While attention should also be paid to identifying other common ADRs, such as HFS and hypertension.

The overall incidence of ADRs (90.2%) in our study is consistent with the GRID multinational pivotal phase 3 clinical trial in GIST, in which 98.5% of regorafenib recipients experienced a drug-related adverse event (7) and also with an observational post-marketing surveillance study in Japanese patients with colorectal cancer (89.3%) (9). In the current study, the most common ADRs were HFS, hypertension, abnormal hepatic function, and malaise. This profile is generally consistent with the adverse events reported in previous studies in patients with GIST (7).

Considering clinical outcome in relation to HFS, OS tended to be better in patients who developed HFS. However, the survival differences between patients with no HFS and those with HFS of any grade were not statistically significant. *Post hoc* analyses of the phase 3 CORRECT and RESORCE studies of regorafenib for colorectal cancer and hepatocellular carcinoma found an association between HFS and improved OS (10, 11), as did a retrospective study in colorectal cancer (12). Additional studies with GIST are needed to further investigate the association between HFS and OS.

In the present study, both OS and TTF were improved in patients whose ECOG performance status was rated as good, similar findings having been reported in patients with colorectal cancer treated with regorafenib (9, 13). Although more than 90% of patients in the current study were previously treated with imatinib or sunitinib, these findings suggest that the initiation of regorafenib in patients with good performance status may improve the outcome, and it may be desirable to avoid administering regorafenib to patients with a poor performance status.

With respect to effectiveness, the DCR in the current study (56.5%) was similar to that reported for recipients of regorafenib in GRID (overall population 52.6%; Japanese subgroup 58%) (2, 7). Patient characteristics in the present study were similar to those of the population in the GRID pivotal clinical trial, except that most patients in the current study received regorafenib as third-line therapy (84.6%) whereas only 56.8% of GRID participants received it as third-line therapy.

Unlike colorectal cancer, where molecular profiling is helping to develop agents targeting specific pathways/biological sites associated with tumors (14, 15), the development of drugs for the treatment of GIST has not progressed to the same extent (4, 16). This is an unmet need in this patient population and until a wider range of alternative treatment options becomes available, it is important to make the best use of therapies available to us. In this regard, regorafenib is an established third-line therapy (17) and we need to continue to provide education to healthcare professionals about its optimal use and measures to prevent or limit potential ADRs associated with its administration. This should also include advice regarding switching from a previous treatment to regorafenib in patients with GIST.

## Conclusion

Outcomes in this real-world study in Japan were consistent with those seen in previous clinical trials. No new safety concerns were identified. To prevent early treatment discontinuation because of ADRs, monitoring adverse events in the early period of treatment with regorafenib is strongly recommended. This study provided promising results in an appropriate selection of patients with advanced GIST in clinical practice including patients with ECOG PS of 0 or 1.

## Data availability statement

The original contributions presented in the study are included in the article/**Supplementary Material**. Further inquiries can be directed to the corresponding author.

## Ethics statement

Ethical approval was not required for the studies involving humans because the study (ClinicalTrials.gov, NCT01933958) was conducted in compliance with the Good Post-Marketing Study Practice (GPSP) and Good Vigilance Practice (GVP) of the Ministry of Health, Labor, and Welfare in Japan. Approval by the ethics committee of each institution was not required because GPSP and GVP do not require such approval for Post Marketing Surveillance (PMS) studies. The studies were conducted in accordance with the local legislation and institutional requirements. Written informed consent for participation was not required from the participants or the participants’ legal guardians/next of kin in accordance with the national legislation and institutional requirements because the study was conducted in compliance with the GPSP and GVP in Japan.

## Author contributions

YK: Writing – review & editing, Writing – original draft, Supervision, Formal analysis, Conceptualization. KM: Investigation, Writing – review & editing, Writing – original draft, Supervision, Conceptualization. MC: Supervision, Writing – review & editing, Writing – original draft, Formal analysis, Conceptualization. KHi: Supervision, Writing – review & editing, Writing – original draft, Formal analysis, Conceptualization. TS: Writing – review & editing, Writing – original draft, Supervision, Formal analysis, Conceptualization. KA: Writing – review & editing, Writing – original draft, Supervision, Formal analysis, Conceptualization. KHa: Writing – review & editing, Writing – original draft, Supervision, Conceptualization. TN: Writing – review & editing, Writing – original draft, Supervision, Investigation, Formal analysis, Conceptualization.
