# Supplementary material for: Large-scale, prospective observational study of regorafenib in Japanese patients with advanced gastrointestinal stromal tumors in a real-world clinical setting
Source: Front Oncol. 2024 Jun 17;14:1412144. doi: 10.3389/fonc.2024.1412144 (PMC11215966; doi:10.3389/fonc.2024.1412144)
Supplement: Supplementary file 1 [file DataSheet_1.docx]

Supplementary Material

Large-scale, prospective observational study of regorafenib in Japanese patients with advanced gastrointestinal stromal tumors in a real-world clinical setting

**Yoshito Komatsu^*^, Kei Muro, Masayuki Chosa, Kazufumi Hirano, Toshiyuki Sunaya, Koichi Ayukawa, Kana Hattori, Toshirou Nishida**

*** Correspondence:** Yoshito Komatsu, M.D.: [ykomatsu@med.hokudai.ac.jp](mailto:ykomatsu@med.hokudai.ac.jp)

**Supplemental Table 1.** Adverse drug reactions (ADRs) according to initial daily dose, for all ADRs and for ADRs of special interest (n=143)

|  | **N** | **Patients who developed ADRs, n (%)** |
| --- | --- | --- |
| All ADRs | 143 | 129 (90.2) |
| 80mg | 34 | 28 (82.4) |
| 120mg | 49 | 43 (87.8) |
| 160mg | 60 | 58 (96.7) |
| Hepatic function disorder ^a^ | 143 | 38 (26.6) |
| 80mg | 34 | 5 (14.7) |
| 120mg | 49 | 10 (20.4) |
| 160mg | 60 | 23 (38.3) |
| Hypertension/hypertensive crisis ^a^ | 143 | 46 (32.2) |
| 80mg | 34 | 11 (32.4) |
| 120mg | 49 | 15 (30.6) |
| 160mg | 60 | 20 (33.3) |
| Hand and foot syndrome ^a^ | 143 | 91 (63.6) |
| 80mg | 34 | 17 (50.0) |
| 120mg | 49 | 31 (63.3) |
| 160mg | 60 | 43 (71.7) |
| Fatigue/Malaise ^b^ | 143 | 24 (16.8) |
| 80mg | 34 | 2 (5.9) |
| 120mg | 49 | 8 (16.3) |
| 160mg | 60 | 14 (23.3) |

^a^ ADR classified as an important research item.

^b^ ADR classified as requiring special attention.

NOTE: ADRs were coded using MedDRA /J (version 24.0).

**Supplemental Figure 1.** Timing of the first occurrence of adverse drug reactions (<= 1, 60, 182, 365, or 547 day(s)): all adverse drug reactions and adverse drug reactions of grade ≥3


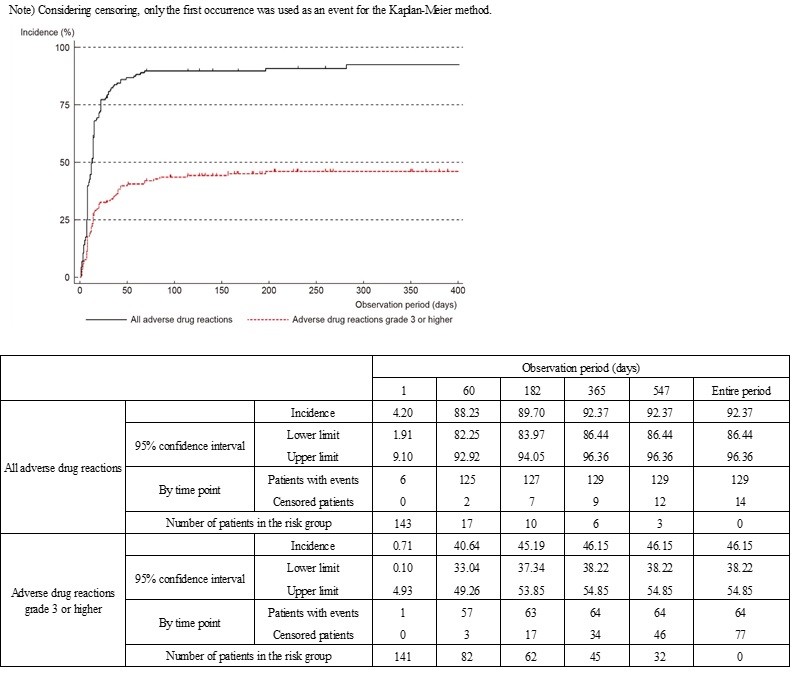


- Period until event or censoring: It is “the date of the first occurrence of the event of interest or the last observation - date of start of first dose + 1 day”. The last observation date (censoring date) for cases in which no event occurred is the bigger value of the last survival observation date or death date in the patient outcome column of the questionnaire.
- The occurrence of Grade ≥3 ADRs in the important identified risks is described below. Hepatic function disorder in 7.69% of patients (11/143 patients), Thromboembolism in 2.80% of patients (4/143 patients), Hypertension/hypertensive crisis in 16.08% of patients (23/143 patients), Hemorrhage in 1.40% of patients (2/143 patients), HFS in 15.38% of patients (22/143 patients), Gastrointestinal perforation and fistulae in 0.70% of patients (1/143 patients), Thrombocytopenia in 1.40% of patients (2/143 patients), Interstitial lung disease in 0.70% of patients (1/143 patients).

**Supplemental Figure 2.** Timing of the occurrence of adverse drug reactions (<= 1, 60, 182, 365, or 547 day(s)): hepatic function disorder/hypertension or hypertensive crisis/hand and foot syndrome/fatigue or malaise


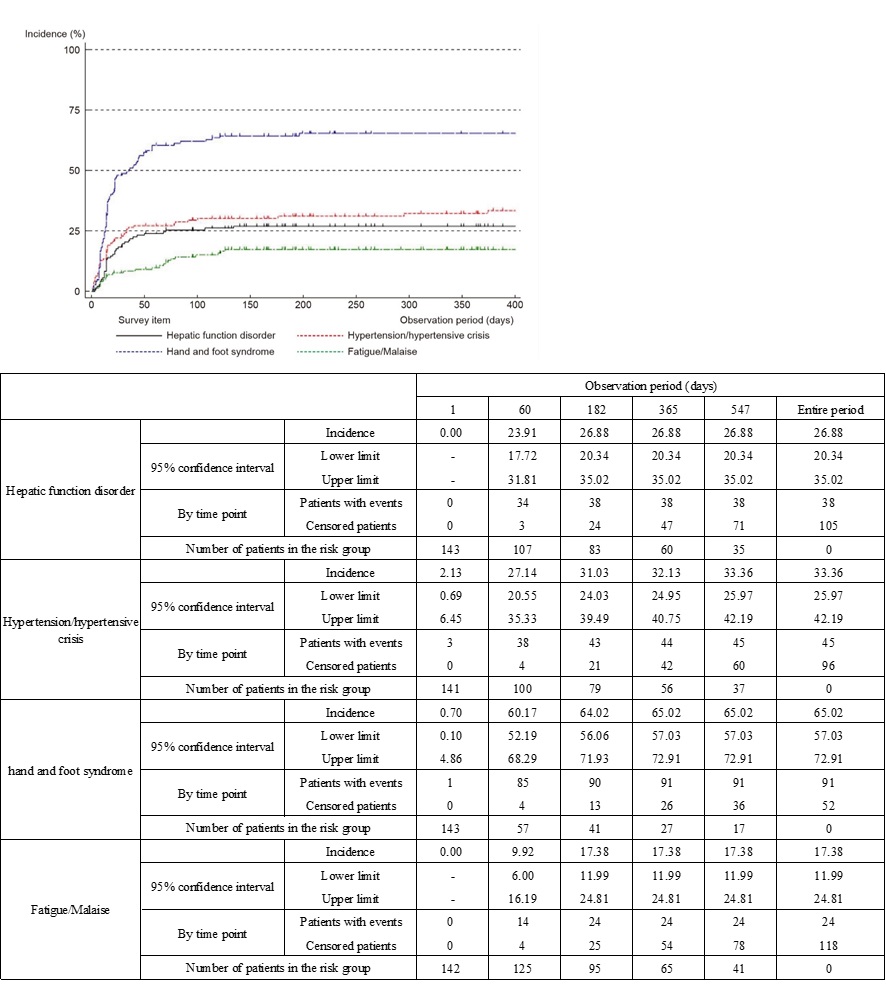


**Supplemental Figure 3.** Timing of the occurrence of adverse drug reactions (<= 1, 60, 182, 365, or 547 day(s)): hepatic function disorder according to initial daily dose


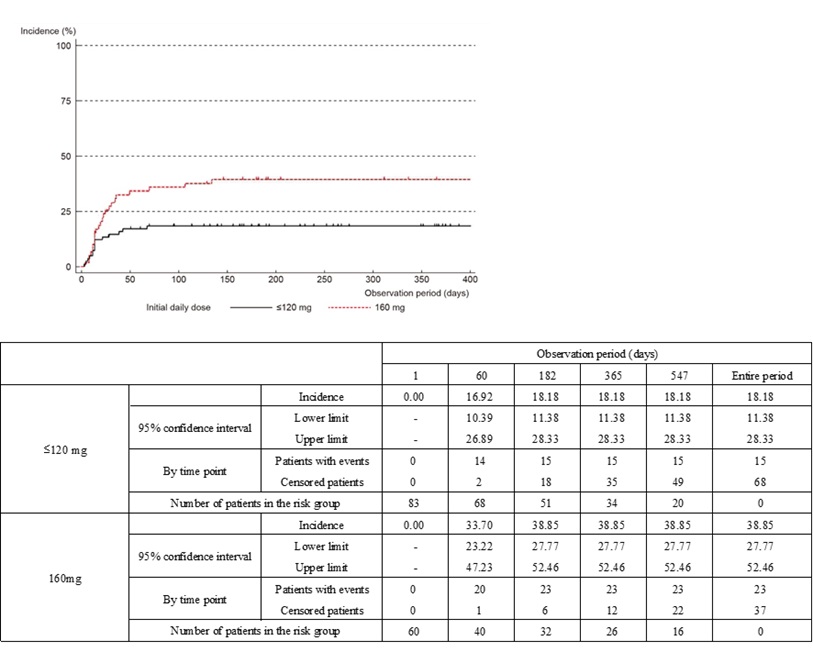


**Supplemental Figure 4.** Timing of the occurrence of adverse drug reactions (<= 1, 60, 182, 365, or 547 day(s)): hypertension or hypertensive crisis according to initial daily dose
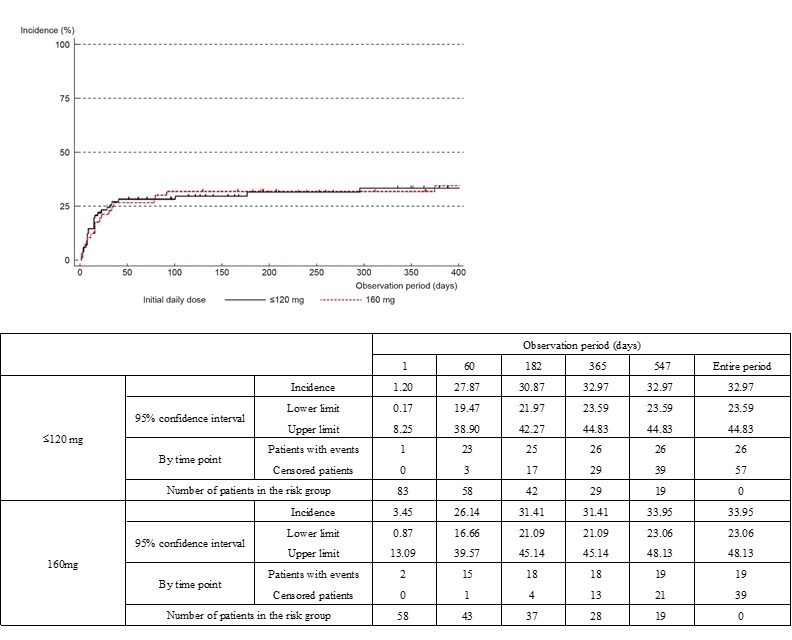


**Supplemental Figure 5.** Timing of the occurrence of adverse drug reactions: hand and foot syndrome according to initial daily dose


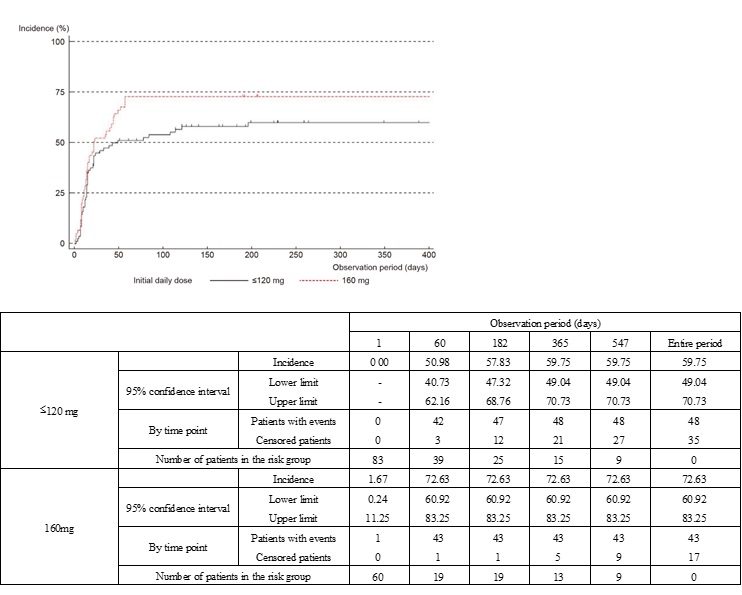


**Supplemental Figure 6.** Timing of the occurrence of adverse drug reactions: fatigue or malaise according to initial daily dose


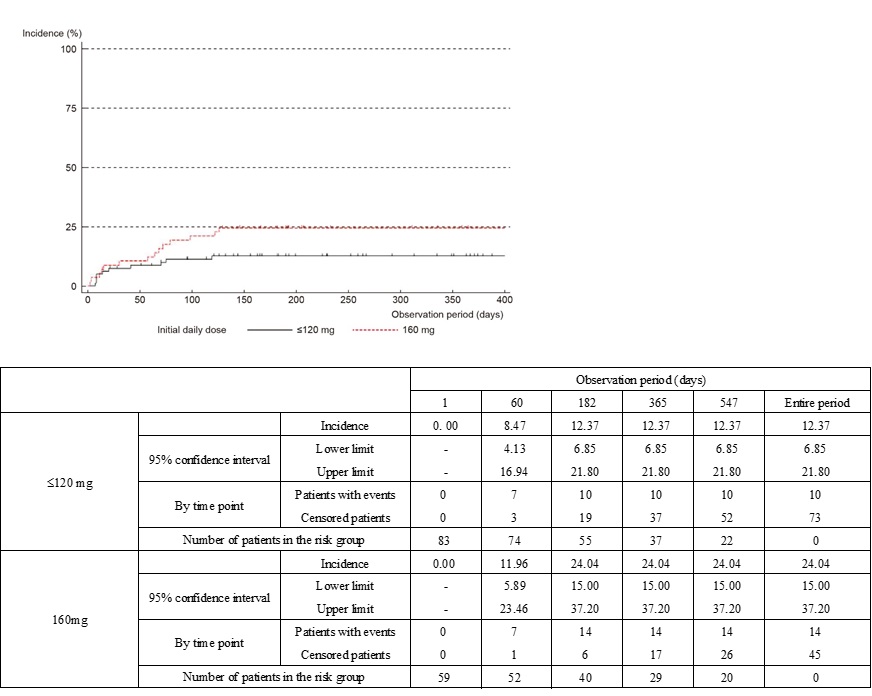


**Supplemental Figure 7.** Overall survival according to baseline ECOG performance status (PS)

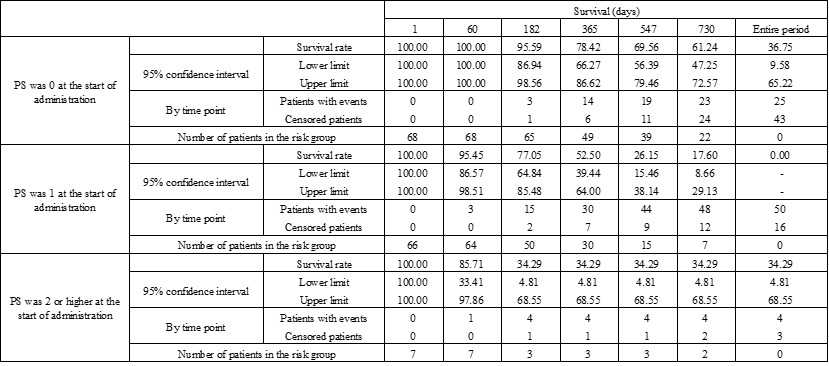


**Supplemental Figure 8.** Time to treatment failure according to baseline ECOG performance status (PS)

**Supplemental Figure 9.** Transition of treatment discontinuation after the start of Regorafenib therapy^1)2)^


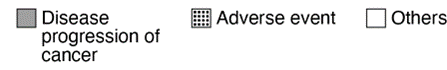

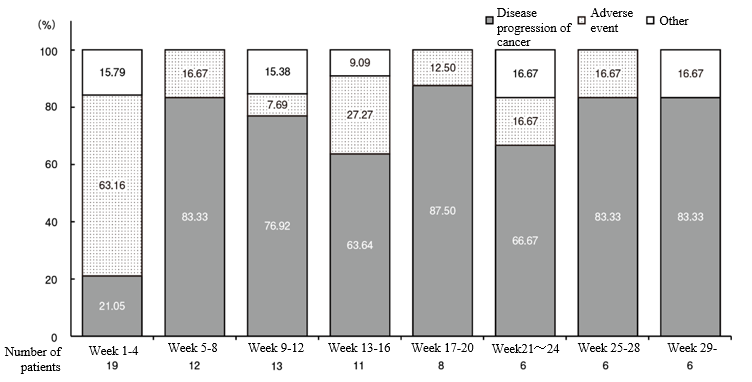


1) The numbers of patients with disease progression of cancer, adverse events, and other are shown as percentages of the patients for each period.

2) The number of patients who were listed for both disease progression of cancer and adverse events were calculated as disease progression of cancer.
